# Supplementary material for: ARID1A loss induces polymorphonuclear myeloid-derived suppressor cell chemotaxis and promotes prostate cancer progression
Source: Nat Commun. 2022 Nov 26;13:7281. doi: 10.1038/s41467-022-34871-9 (PMC9701216; doi:10.1038/s41467-022-34871-9)
Supplement: Supplementary file 1 — Supplementary Information [file 41467_2022_34871_MOESM1_ESM.pdf]

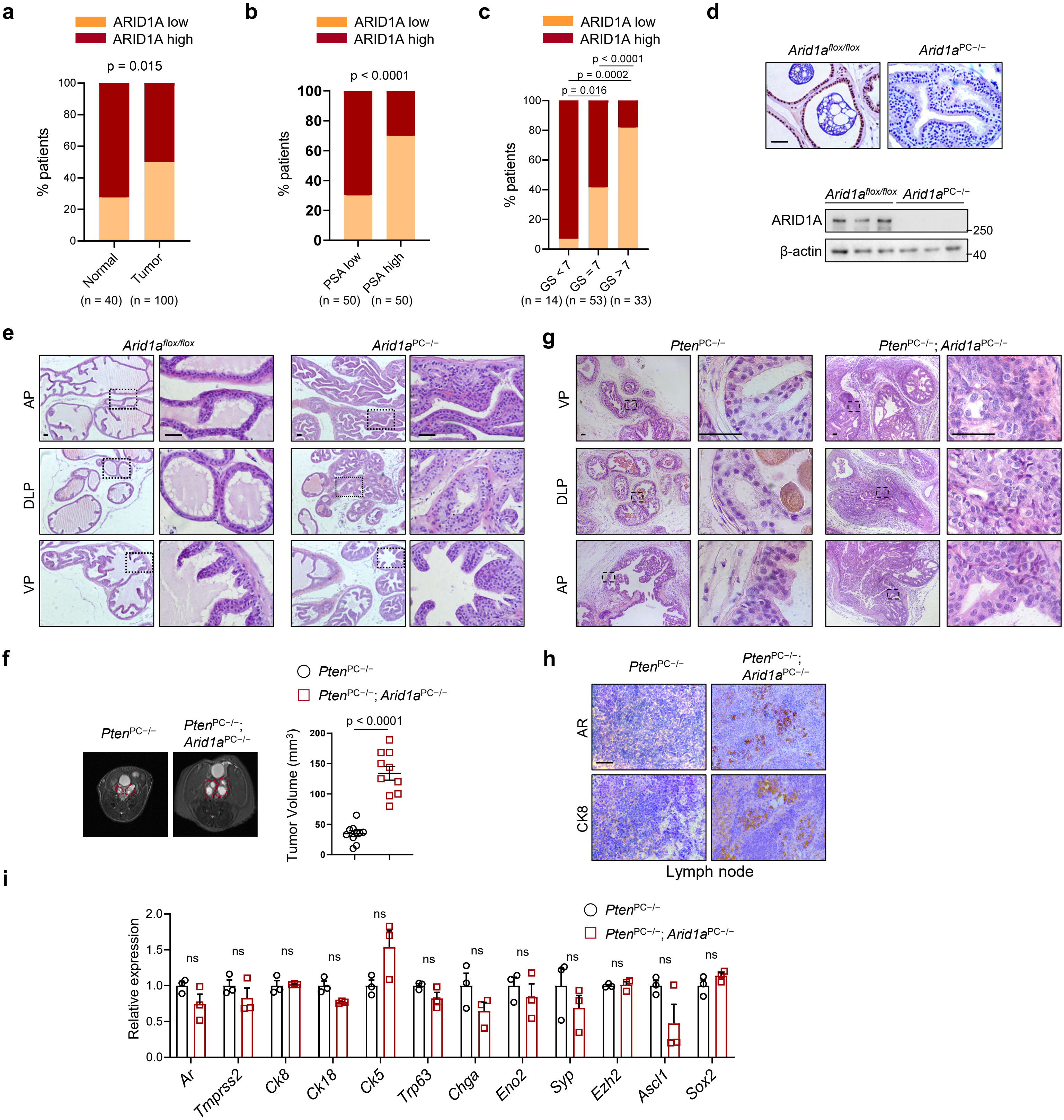

Supplementary Figure 1. *Arid1a* inactivation promotes prostate tumorigenesis.

a, ARID1A staining indexes using a 10-point quantification scale in cohorts of normal prostate tissues (n = 40) and tumors (n = 100). **b**, ARID1A expression levels stratified by PSA in TMA (n = 100). **c**, IHC score of ARID1A stratified by Gleason score (GS) in TMA (n = 100). **d**, ARID1A-stained sections of representative DLP and IB analysis of tissues from 6-month-old *Arid1a*$^{\text{flox/flox}}$ and *Arid1a*$^{\text{PC}-/-}$ mice (n = 3). Scale bar: 50 µm. **e**, H&E-stained sections of representative AP, DLP, and VP in mice at 12 months of ages (n =10, representative data are shown). Scale bar: 50 µm. **f**, MRI analysis and quantitation of 16-week-old *Pten*$^{\text{PC}-/-}$ and *Pten*$^{\text{PC}-/-}$; *Arid1a*$^{\text{PC}-/-}$ prostates (n = 10). **g**, H&E-stained sections of 12-week-old *Pten*$^{\text{PC}-/-}$ and *Pten*$^{\text{PC}-/-}$; *Arid1a*$^{\text{PC}-/-}$ mice (n = 15, representative data are shown). Scale bars, 50 µm. **h**, AR and CK8 staining of lymph nodes from 12-week-old mice. Scale bar, 50 µm. **i**, Relative expression of lineage markers in epithelium from 12-week-old mouse prostates (n = 3), ns, no significance.

f, **i**, Data represent the mean ± SEM. Statistical significance was determined by two-tailed χ^2 test (**a**, **b**, **c**) and two-tailed unpaired *t* test (**f**, **i**). Source data are provided as a Source Data file.

Supplementary Figure 2

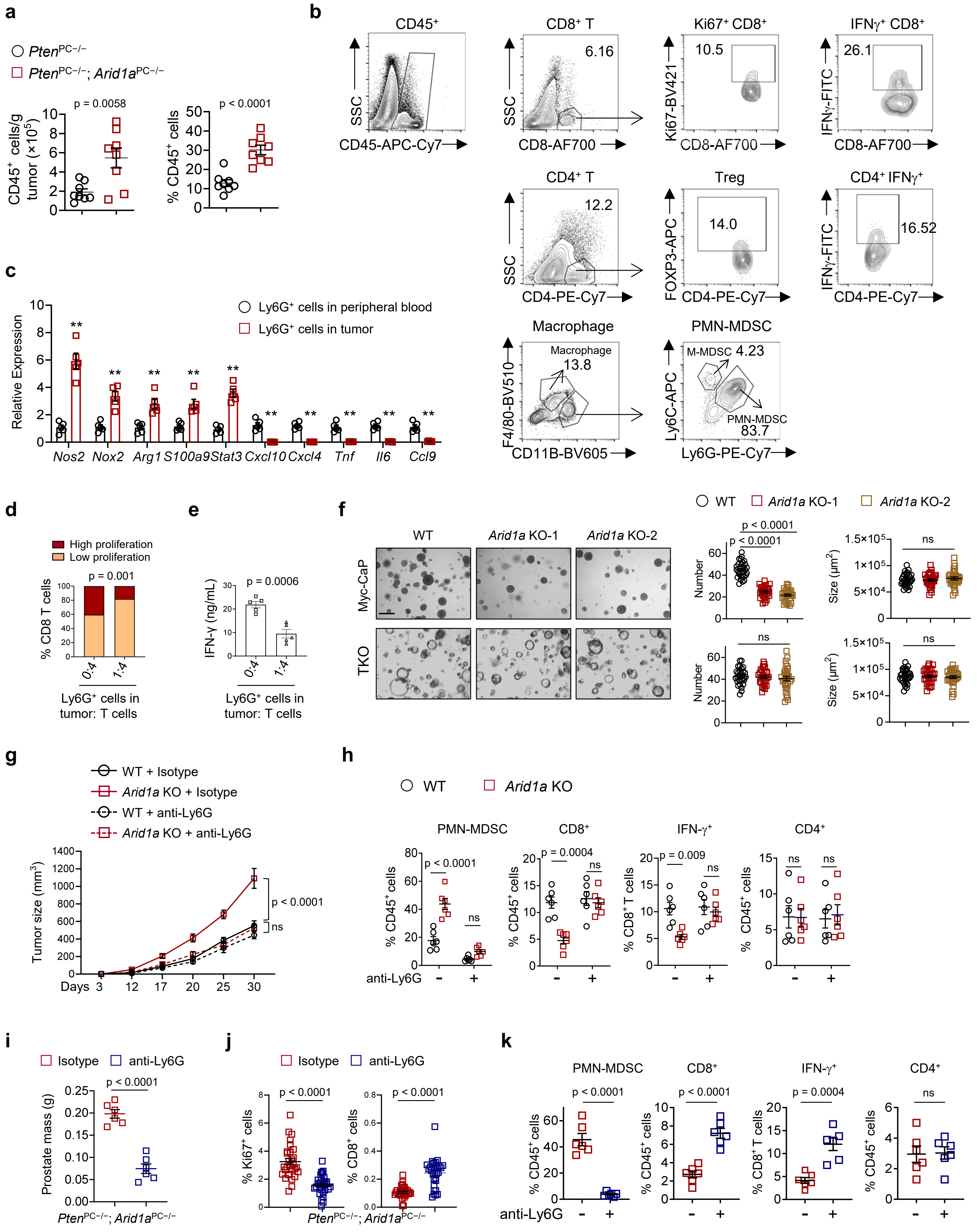

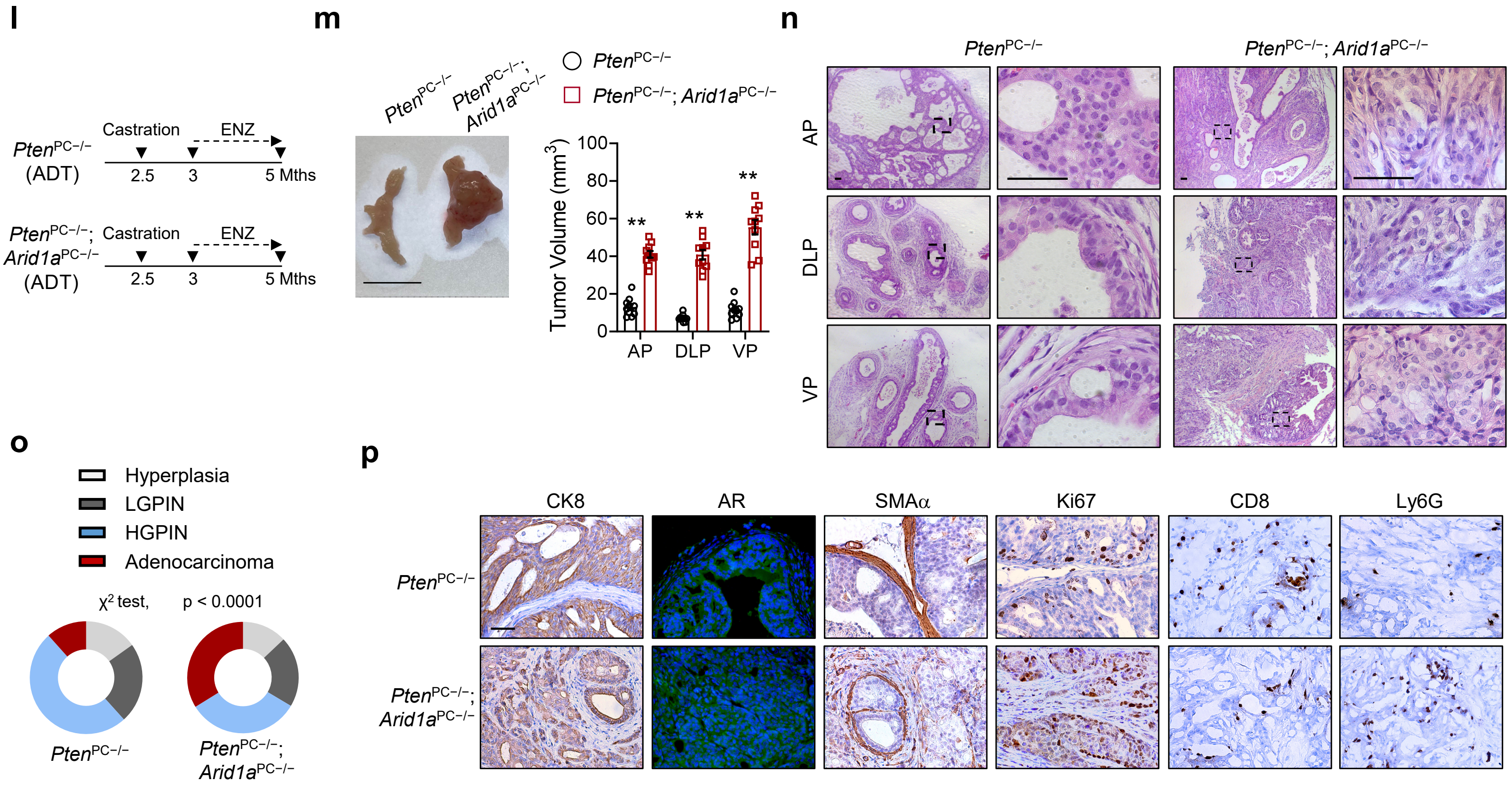

Supplementary Figure 2. PMN-MDSC enrichment promotes the progression of *Arid1a*-deleted tumors.
a, Percentage of $CD45^+$ immune cells in 3-month-old *Pten*$^{PC-/-}$ and *Pten*$^{PC-/-}$; *Arid1a*$^{PC-/-}$ prostate tumors (n = 8). **b**, Gating strategy for FACS analysis (Fig. 2c) is shown for tumor-infiltrating immune cells. The same strategy was used for the subsequent experiments. **c**, qRT-PCR analysis of $Ly6G^+$ cells from 3-month-old *Pten*$^{PC-/-}$; *Arid1a*$^{PC-/-}$ prostate tumors and peripheral blood of WT mice (n = 5). *p* values for the individual gene expression in $Ly6G^+$ cells of peripheral blood and tumor: *Nos2* ($p < 0.0001$), *Nox2* ($p = 0.0004$), *Arg1* ($p = 0.0017$), *S100a9* ($p = 0.0019$), *Stat3* ($p < 0.0001$), *Cxcl10* ($p = 0.0001$), *Cxcl4* ($p < 0.0001$), *Tnf* ($p = 0.0001$), *Il6* ($p < 0.0001$), *Ccl9* ($p = 0.0001$). **d**, Summary of T cell proliferation assessed by CFSE flow cytometry after 4 days of co-culture. High and low proliferation were defined as T cell division ≥ 2 and ≤ 1, respectively (n = 5). **e**, IFN-γ secretion by $CD8^+$ T cells measured by ELISA (n = 5). **f**, Representative images of organoids and quantification as indicated (n = 10 fields from 3 experiments per group). Scale bar, 500 µm. **g**, Volume of subcutaneous tumors derived from WT and *Arid1a* KO Myc-CaP cells with or without anti-Ly6G antibody treatment. WT + Isotype, WT + anti-Ly6G or *Arid1a* KO + anti-Ly6G (n = 12, each group); *Arid1a* KO + Isotype (n = 11). **h**, Quantification of tumor-infiltrating immune cells in WT and *Arid1a* KO Myc-CaP subcutaneous tumors with or without anti-Ly6G antibody treatment (n = 6). **i**, The prostate weights of *Pten*$^{PC-/-}$; *Arid1a*$^{PC-/-}$ mice with or without anti-Ly6G administration. The treatment was performed twice a week for 4 weeks when the mice were 10-week-old (n = 6). **j**, Quantification of $Ki67^+$ and $CD8^+$ in prostate sections. **k**, Flow cytometry analysis of PMN-MDSCs, $CD8^+$ T cells and $IFN\gamma^+$ $CD8^+$ T cells (n = 6). **l**, Graphical scheme describing the ADT strategy. **m**, Gross photographs and volume of prostates from the indicated mice with ADT (n = 10). Scale bar, 1 cm. *p* value for the prostatic volume: AP ($p < 0.0001$), DLP ($p < 0.0001$) and VP ($p < 0.0001$). **n**, H&E-stained sections in 5-month-old *Pten*$^{PC-/-}$ and *Pten*$^{PC-/-}$; *Arid1a*$^{PC-/-}$ prostates after ADT (n = 10, representative data are shown). Scale bars, 50 µm. **o**, Quantification of histological grade from the indicated mice with ADT (n = 10). **p**, Immunohistochemical analysis in castrated prostate sections. Scale bar, 50 µm.
a, **c**, **e**-**k**, **m**, Data represent the mean ± SEM. Statistical significance was determined by two-tailed unpaired *t* test (**a**, **c**, **e**, **i**-**k**, **m**), Fisher's exact test (**d**), one-way ANOVA followed by multiple comparisons (**f**), two-way ANOVA followed by multiple comparison (**g**, **h**) and χ^2 test (**o**) . **p**, Representative data of triplicate experiments are shown. Source data are provided as a Source Data file. ***p* < 0.01, ns, no significance.

Supplementary Figure 3

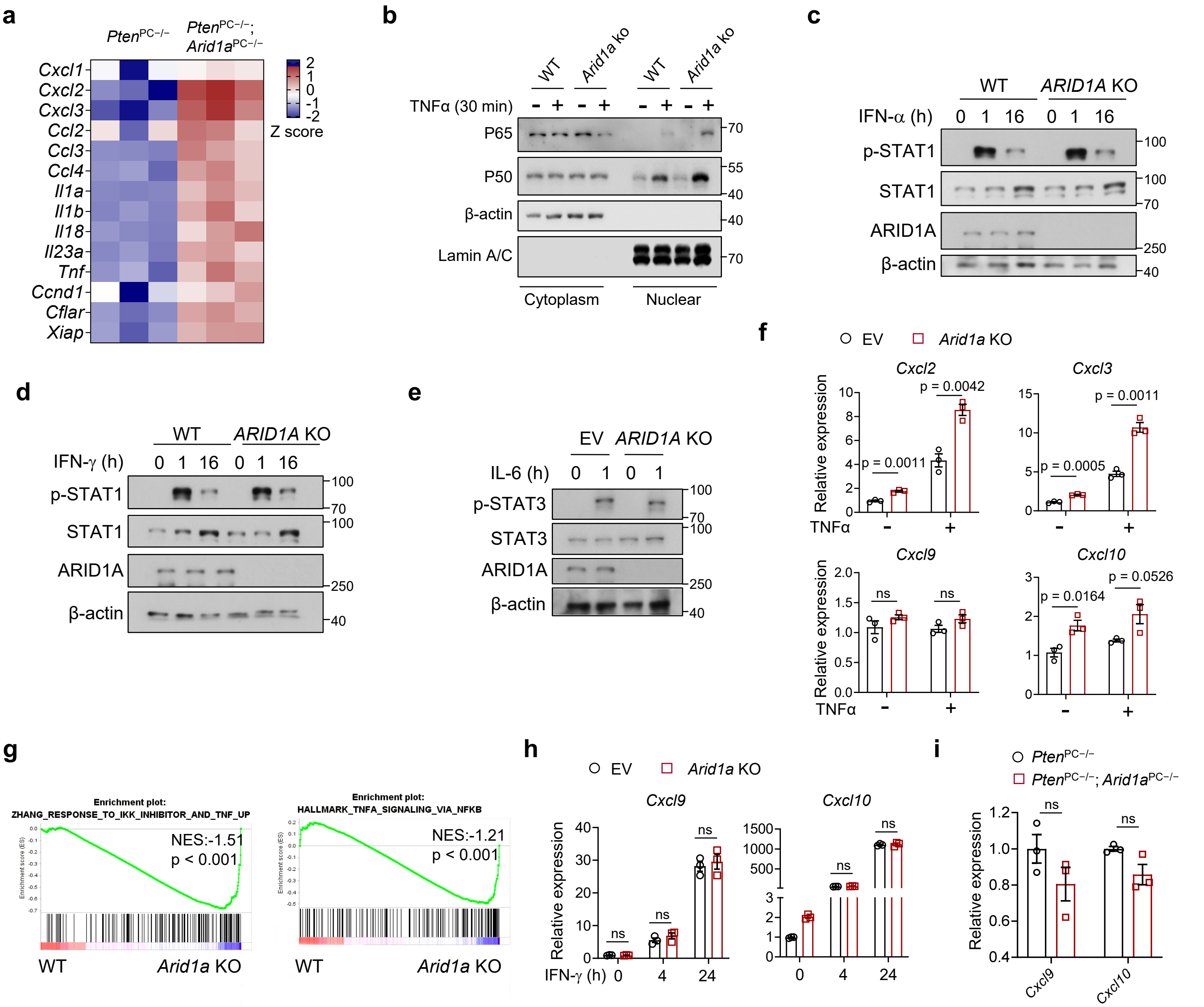

Supplementary Figure 3. ARID1A loss activates NF-κB signaling to enhance the recruitment of MDSCs.

a, Heatmap summarizing the qRT-PCR results in epithelial cells of 3-month-old *Pten*$^{PC-/-}$ and *Pten*$^{PC-/-}$; *Arid1a*$^{PC-/-}$ mouse prostates (n = 3). **b**, IB analysis of the indicated proteins in cytoplasmic extracts and nuclear extracts from Myc-CaP cells stimulated with TNFα (30 min). **c-e**, IB analysis of WT and *Arid1a*-KO Myc-CaP cells with or without IFNα (**c**), IFNγ (**d**) and IL-6 (**e**) stimulation. **f**, The qRT-PCR results in WT and *Arid1a* KO Myc-CaP cells with or without TNFα stimulation (n =3). **g**, Hyperactivation of NF-κB in *Arid1a*-depleted Myc-CaP cells analyzed by GSEA. NES, normalized enrichment score. *p* value was determined by GSEA. **h**, *Cxcl9* and *Cxcl10* transcripts were quantified by real-time PCR in sg*Arid1a*- and vector-expressing Myc-CaP cells stimulated with IFNγ at the indicated time points (n = 3). **i**, *Cxcl9* and *Cxcl10* transcripts were quantified by real-time PCR in epithelial cells of 3-month-old *Pten*$^{PC-/-}$ and *Pten*$^{PC-/-}$; *Arid1a*$^{PC-/-}$ prostates (n = 3).

f, **h**, **i**, Data represent the mean ± SEM and statistical significance was determined by two-tailed unpaired *t* test. **b-e**, Data were evaluated in triplicate, and representative data are shown. Unprocessed immunoblots are shown as source data. ns, no significance.

Supplementary Figure 4

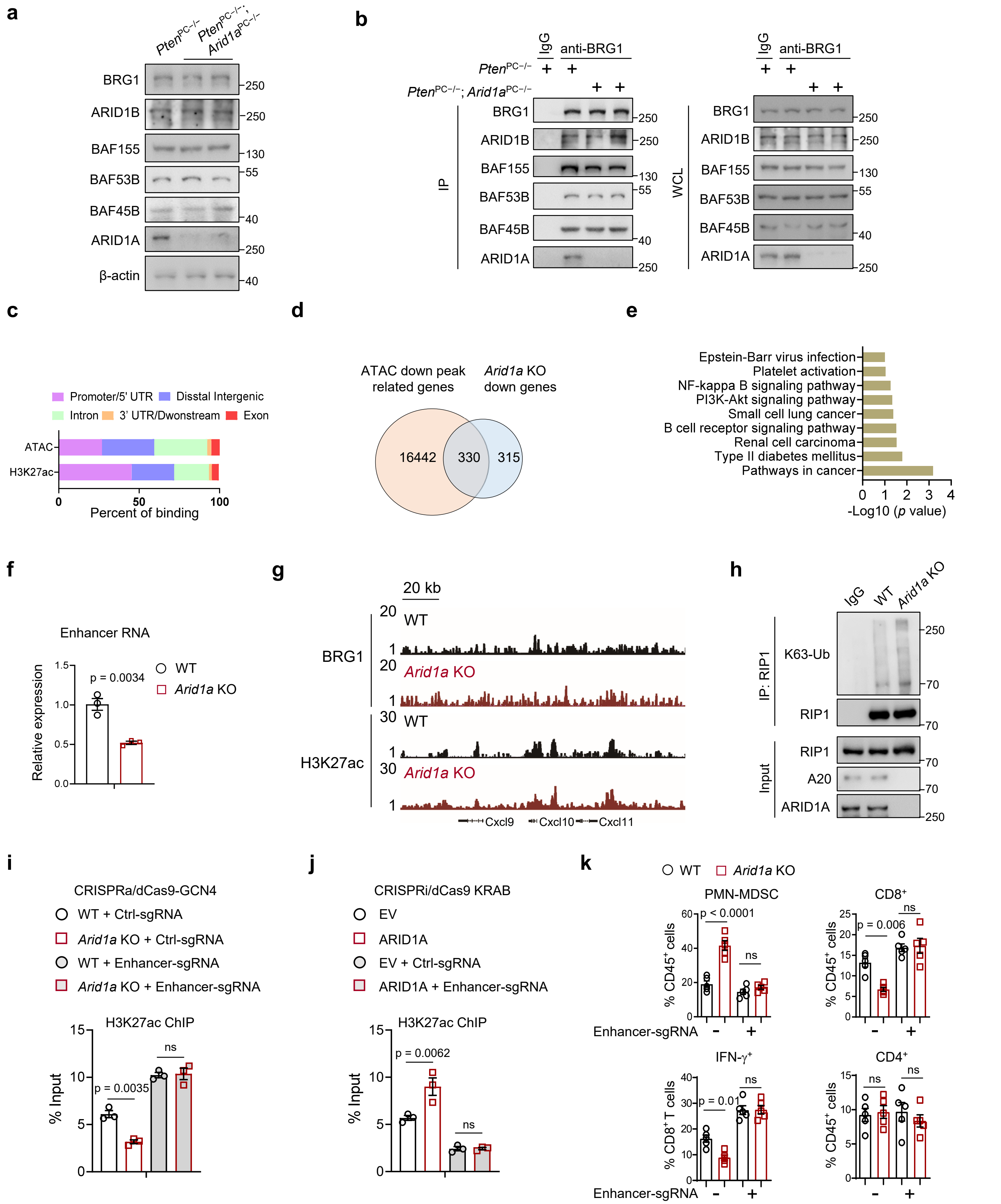

Supplementary Figure 4. *Arid1a* ablation silences the enhancer of the A20 gene to stimulate NF-κB signaling. **a**, IB analysis of the indicated protein in 3-month-old *Pten*$^{PC-/-}$ and *Pten*$^{PC-/-}$; *Arid1a*$^{PC-/-}$ mouse prostates. **b**, IB analysis of the whole-cell lysate (WCL) from 12-week-old *Pten*$^{PC-/-}$ and *Pten*$^{PC-/-}$; *Arid1a*$^{PC-/-}$ prostates and anti-BRG1 immunoprecipitates as indicated. **c**, Bar charts revealing the distributions of ATAC-seq peaks and H3K27ac ChIP-seq peaks in vector-expressing Myc-CaP cells. **d**, Venn diagram of the genes showing the reduced accessibility and expression simultaneously in *Arid1a* KO Myc-CaP cells compared to WT cells. **e**, GO-BP enrichment of the overlapped 85 genes by DAVID, ranked according to *p* value. **f**, *A20* enhancer RNA were quantified by real-time PCR (n = 3). **g**, ChIP-seq tracks of BRG1 and H3K27ac signals in *Cxcl9*, *Cxcl10* and *Cxcl11* gene loci, as indicated. **h**, IB analyses of the WCL and anti-RIP1 immunoprecipitates of Myc-CaP cells. Cells were treated with 20 ng/ml TNFα for 30 min before harvesting. **i**, ChIP-qPCR assays of H3K27ac at the *A20* enhancer in WT and *Arid1a* KO Myc-CaP cells with or without *A20* enhancer activation (CRISPRa/dCas9-based induction, n = 3). **j**, ChIP-qPCR assays of H3K27ac at the *A20* enhancer in WT and ARID1A overexpressing Myc-CaP cells with or without A20 enhancer silencing (CRISPRi/dCas9-based suppression, n = 3). **k**, Quantification of tumor-infiltrating immune cells in WT and *Arid1a* KO tumors with or without A20 enhancer activation by FACS (n = 5). **f**, **i-k**, Data represent the mean ± SEM and statistical significance was determined by two-tailed unpaired *t* test. **a**, **b**, **h**, Experiments were repeated three times independently with similar results; data from one representative experiment are shown. Source data are provided as a Source Data file. ns, no significance.

Supplementary Figure 5

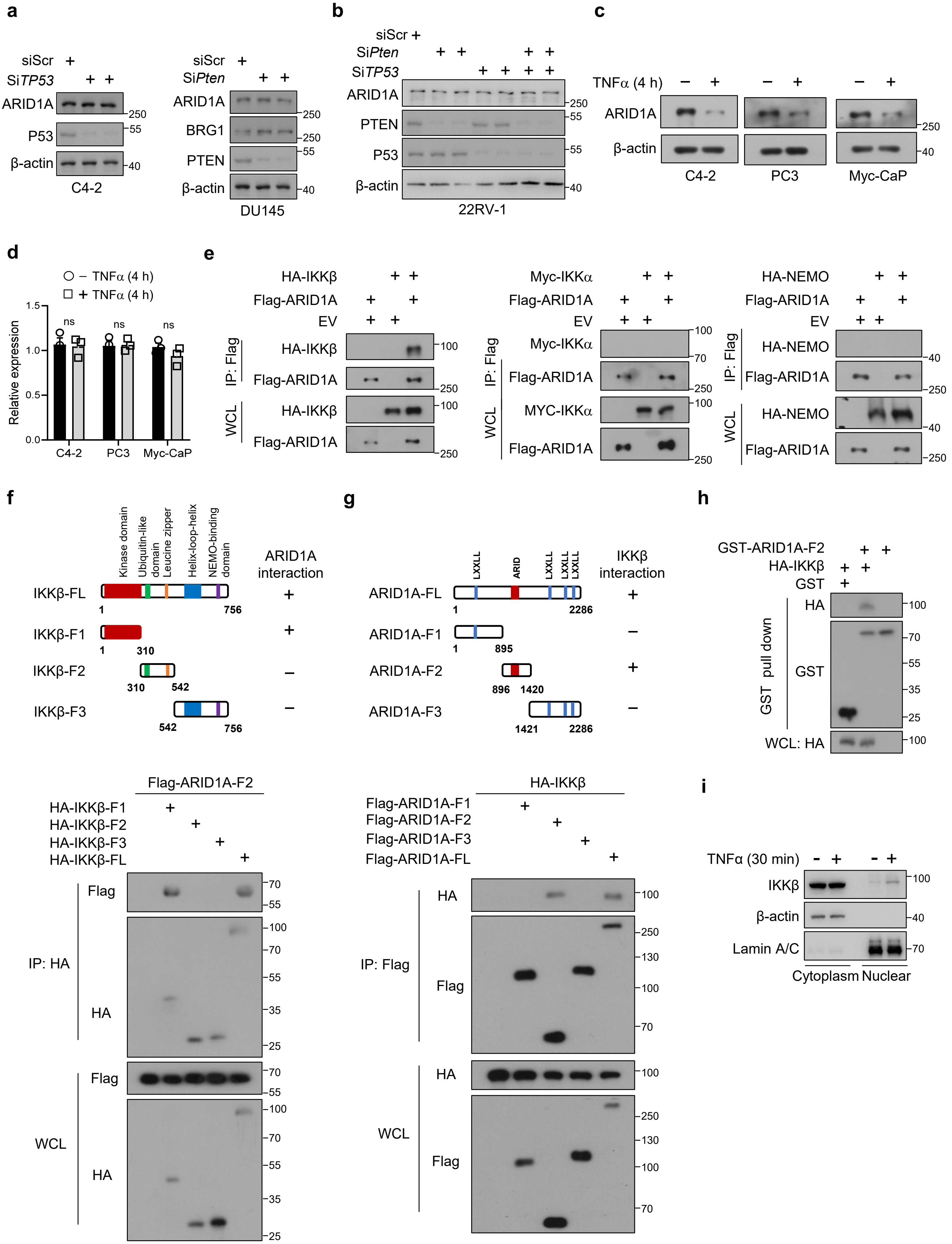

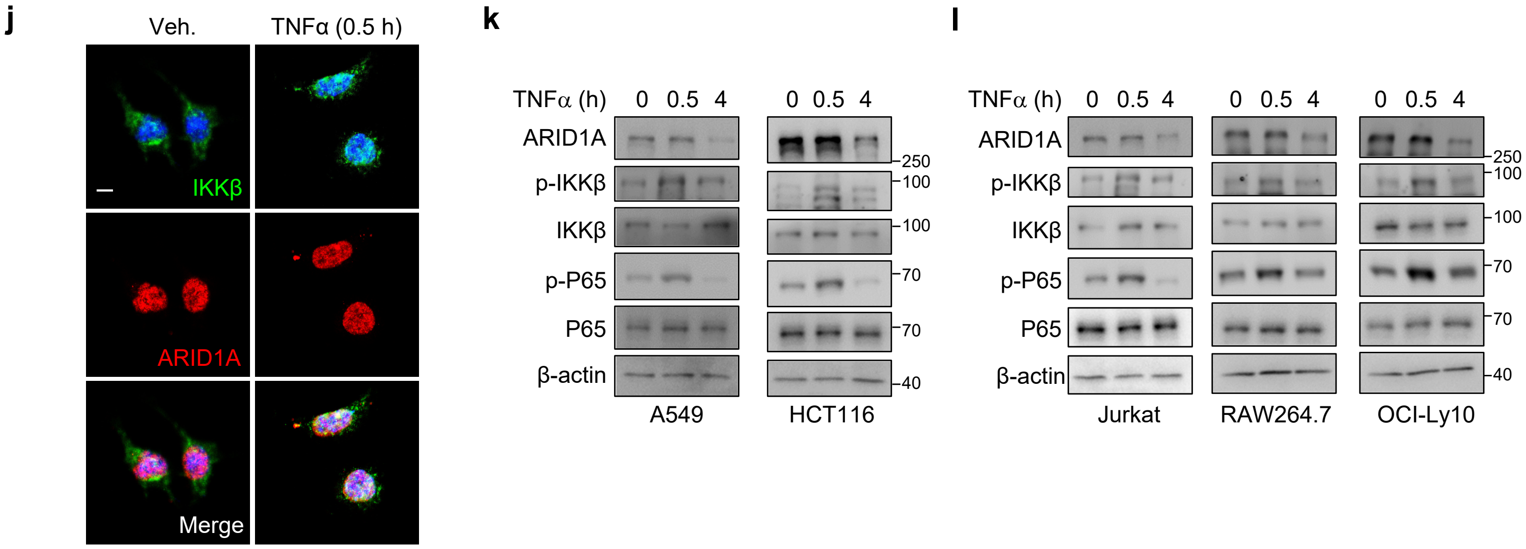

Supplementary Figure 5. IKKβ promotes ARID1A reduction in PCa cells.

a, IB analysis of C4-2 and DU145 cells with *TP53* or *Pten* KD, respectively. **b**, IB analysis of 22RV-1 cells with *TP53* an/or *Pten* KD. **c**, IB analysis of C4-2, PC3 and Myc-CaP cells treated with TNFα for the indicated duration of time. **d**, *ARID1A* transcripts were quantified by real-time PCR in PCa cells with or without TNFα stimulation (n = 3), ns, no significance. **e**, IB analysis of the indicated protein in the WCL and immunoprecipitates from 293T cells transfected with Flag-ARID1A and HA-IKKβ, Myc-IKKα or HA-NEMO. **f**, Interaction between ARID1A and IKKβ. Flag-ARID1A-F2 and HA- IKKβ full-length (IKKβ-FL) and mutants (IKKβ-F1, IKKβ-F2, IKKβ-F3) were ectopically expressed in 293T cells, followed by anti-HA immunoprecipitation and immunoblotting with Flag and HA antibodies. **g**, HA-IKKβ and Flag-ARID1A full-length (ARID1A-FL) and mutants (ARID1A-F1, ARID1A-F2, ARID1A-F3) were ectopically expressed in 293T cells, followed by anti-Flag immunoprecipitation and immunoblotting. **h**, In vitro binding of ARID1A-F2 to IKKβ by GST pulldown analysis. **i**, IB analysis of the indicated proteins in cytoplasmic extracts and nuclear extracts from Myc-CaP cells stimulated with TNFα. **j**, IKKβ and ARID1A immunostaining as indicated in Myc-CaP cells. Scale bar, 100 μm. **k**, IB analysis of A549 and HCT116 cells treated with TNFα for the indicated duration of time. **l**, IB analysis of Jurkat, RAW264.7 and OCI-Ly10 cells treated with TNFα for the indicated duration of time.

d, Data represent the mean ± SEM and statistical significance was determined by two-tailed unpaired *t* test. All experiments were repeated three times independently with similar results; data from one representative experiment are shown. Source data are provided as a Source Data file.

Supplementary Figure 6

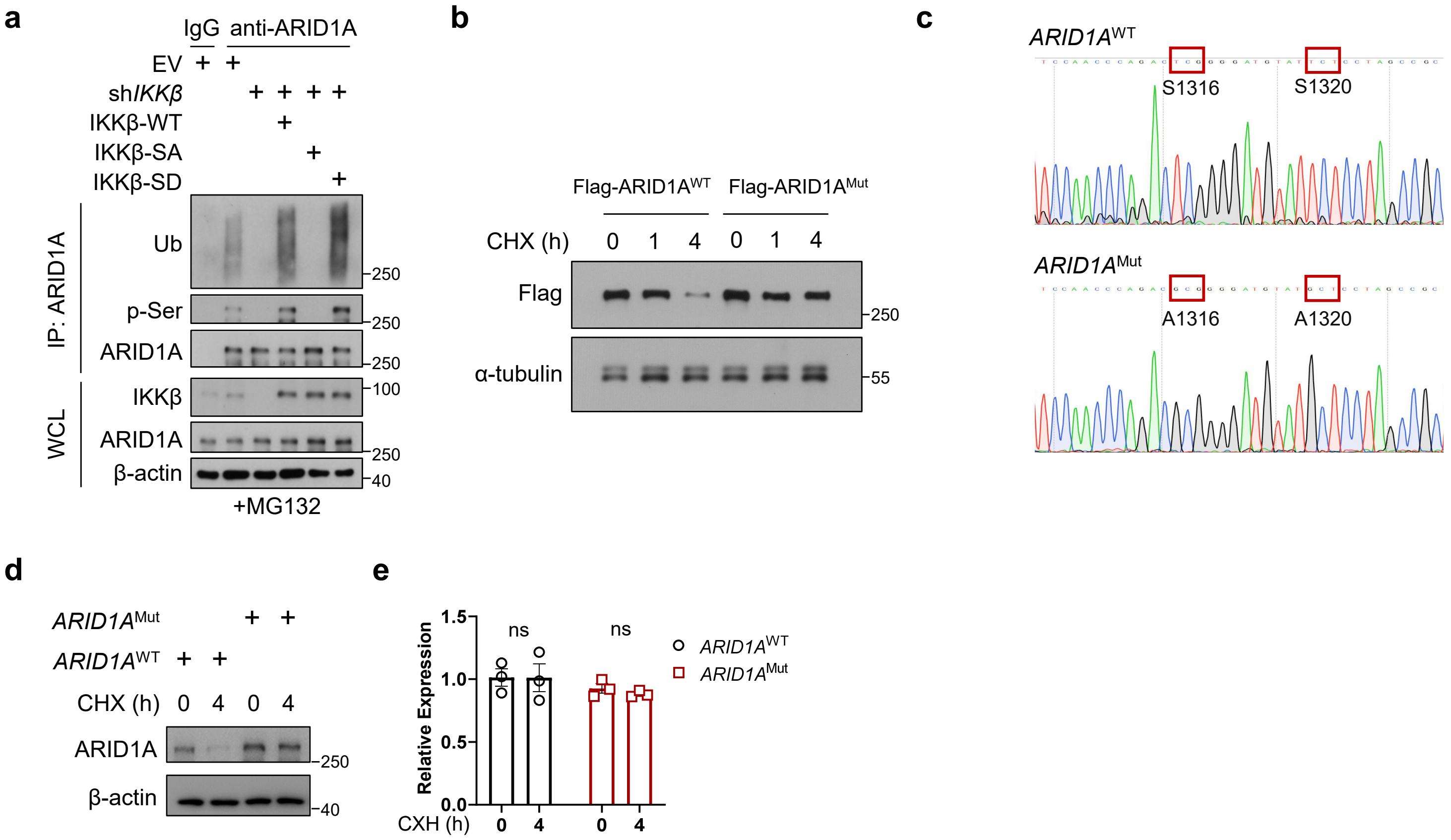

Supplementary Figure 6. IKKβ phosphorylates ARID1A and promotes its β-TRCP-dependent degradation.

a, IB analysis of the WCL and immunoprecipitates of the indicated proteins in C4-2 control and *IKKβ* KD cells with or without IKKβ-WT, IKKβ-SA or IKKβ-SD overexpression. **b**, IB analysis of 293T cells with WT or mutant ARID1A overexpression treated with TNFα for the indicated duration of time. **c**, Sequencing validations of the CRISPR ARID1A-SA knock-in allele in C4-2 cells. **d**, IB analysis of WT and *Arid1a*^{mut} Myc-CaP cells with or without CHX treatment for the indicated duration of time. **e**, *Arid1a* transcripts were quantified by real-time PCR. ns, no significance.

e, Data represent the mean ± SEM and statistical significance was determined by two-tailed unpaired *t* test. **a**, **b**, **d**, Experiments were repeated three times independently with similar results; data from one representative experiment are shown. Source data are provided as a Source Data file.

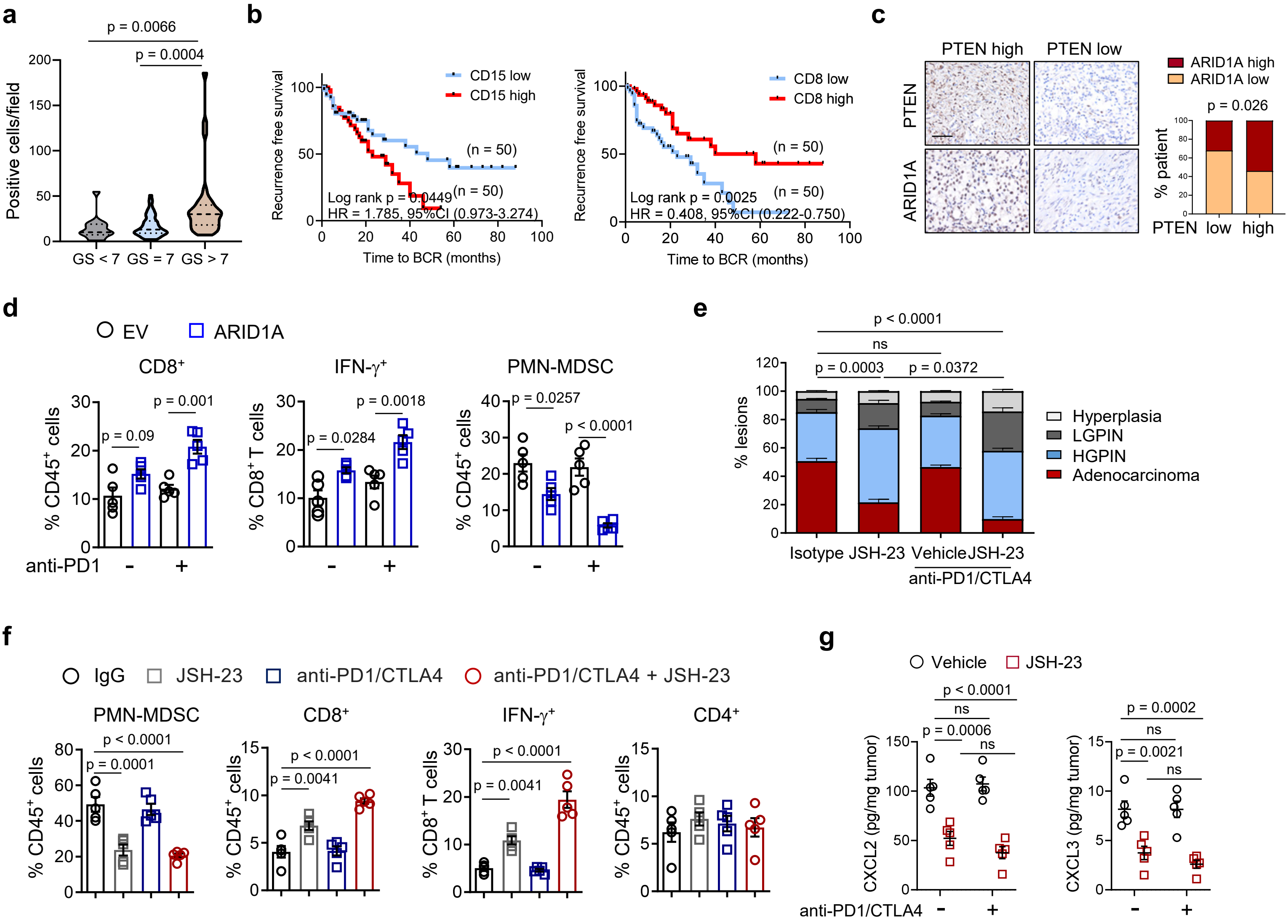

Supplementary Figure 7. Targeting ARID1A or its coordinated signals increase the sensitivity to ICB therapy.

a, CD15 expression levels stratified by GS in TMA (GS < 7, n = 14, GS = 7, n = 53, GS > 7, n = 33). **b**, Kaplan-Meier plot of recurrence after radical prostatectomy based on the proportions of $CD15^+$ and $CD8^+$ cells in patients (n = 50 for each group). **c**, IHC analysis for ARID1A and PTEN. Scale bars, 50 µm. The correlation between PTEN and ARID1A expression is shown as stacked columns (n = 100). **d**, Quantification of each tumor-infiltrating immune cell population in WT and ARID1A-overexpressing Myc-CaP xenografts with or without anti-PD-1 treatment (n = 5). **e**, Prostate tumor histology of *Pten*$^{PC-/-}$; *Arid1a*$^{PC-/}$ mice with or without NF-κB inhibition (JSH-23) in combination with anti-PD1/CTLA-4 treatment (n = 10). **f**, Quantification of the indicated tumor-infiltrating immune cell population of *Pten*$^{PC-/-}$; *Arid1a*$^{PC-/}$ mouse prostates with or without JSH-23 treatment in combination with anti-PD1/CTLA-4 therapy (n = 5). **g**, ELISA of CXCL2 and CXCL3 in prostate tumors of 4-month-old *Pten*$^{PC-/-}$; *Arid1a*$^{PC-/-}$ mice treated as indicated (n = 5).

d-g, Data represent the mean ± SEM. Statistical significance was determined by one-way ANOVA followed by multiple comparisons (**a**), log-rank test (**b**), χ^2 test (**c**, **e**) and two-way ANOVA followed by multiple comparisons (**d**, **f**, **g**). Source data are provided as a Source Data file. ns, no significance.

Supplementary Table 1. Clinical Summary of Tissue Microarray from PCa Patients, Related to Figure 1 and Figure 7

Variables	tumor	Benign Prostatic Hyperplasia
Numbers	100	40
Age at diagnosis, yr	50-82 (mean 67.8)	
Year of surgery	2012/8-2020/3	
PSA at diagnosis, ng/ml	0.48-157.9 (mean 25.4)	
Pathologic Gleason score, n (%)		
6	14 (14)	
7	53 (53)	
8	14 (14)	
9	18 (18)	
10	1 (1)	
No. of biochemical recurrence, n (%)	39 (39)	
Adverse pathologic events, n (%)		
Extra-prostaticextension	18 (18)	
Seminal vesicle invasion	15 (15)	
Positive surgical margins	2 (2)	

Supplementary Table 2. Clinical Summary of Tumor Tissue Specimens from Advanced PCa Patients, Related to Figure 7

Variables	Adenocarcinoma
Numbers	42
Age at diagnosis, yr	49-79 (mean 68)
PSA at diagnosis, ng/ml	0.07-359.87 (mean 58.25)
Pathologic Gleason score at initial diagnosis, n (%)	
8	10 (23.8)
9	28 (66.7)
10	4 (9.5)
Samples from body sites, n (%)	Prostate, 42 (100%)

Supplementary Table 3. Oligonucleotides

Oligonucleotides	
siP50-1	CGCCUGAAUCAUUCUCGAU
siP50-2	CCAGAGUUUACAUCUGAUG
siP52-1	ACAUUGAGGUUCGGUUCUA
siP52-2	GGACAUGACUGCCCAAUUU
siP65-1	CGGAUUGAGGAGAAACGUA
siP65-2	AUGGAUUCAUUACAGCUUA
siIKKα-1	GCAGAUGACGUAUGGGAUA
siIKKα-2	UAGGGUCUGGGAUUCGAUA
siIKKβ-1	CCAGCCAAGAAGAGUGAAG
siIKKβ-2	GCUGGUUCAUAUCUUGAAC
siNEMO-1	GAGGGAGUACAGCAAACUG
siNEMO-2	GCUCGAUCUGAAGAGGCAG
siPTEN-1	GCAGAUAAUGACAAGGAAUAUUA
siPTEN-2	GGUGAAGAUAUAUUCCUCCAAUA
siTRP53-1	GGAGTATTTGGATGACAG
siTRP53-2	AACCUCUUGGUGAACCUUAGUAC
**Primers for shRNA**	
ARID1A-shRNA-homo-1	F:CCGGGCCTGTGCAGTAGAGTGTAGACTCGAGTCTACACTCTACTGCACAGGCTTTTTG
	R:AATTCAAAAAGCCTGTGCAGTAGAGTGTAGACTCGAGTCTACACTCTACTGCACAGGC
ARID1A-shRNA-homo-2	F:CCGGGCTGCCACGTGTGTATATATACTCGAGTATATATACACACGTGGCAGCTTTTTG
	R:AATTCAAAAAGCTGCCACGTGTGTATATATACTCGAGTATATATACACACGTGGCAGC
ARID1A-shRNA-homo-3	F:CCGGGCGAGACACAGCTATTTAATCCTCGAGGATTAAATAGCTGTGTCTCGCTTTTTG
	R:AATTCAAAAAGCGAGACACAGCTATTTAATCCTCGAGGATTAAATAGCTGTGTCTCGC
IKKβ-shRNA-homo-1	F:CCGGGCTGATTGTGTGTGTGAAACACTCGAGTGTTTCACACACACAATCAGCTTTTTG
	R:AATTCAAAAAGCTGATTGTGTGTGTGAAACACTCGAGTGTTTCACACACACAATCAGC
IKKβ-shRNA-homo-2	F:CCGGGGACAGTGTCCAATTCAAATCCTCGAGGATTTGAATTGGACACTGTCCTTTTTG
	R:AATTCAAAAAGGACAGTGTCCAATTCAAATCCTCGAGGATTTGAATTGGACACTGTCC
IKKβ-shRNA-homo-3	F:CCGGGGATTCAGCTTCTCCTAAACACTCGAGTGTTTAGGAGAAGCTGAATCCTTTTTG
	R:AATTCAAAAAGGATTCAGCTTCTCCTAAACACTCGAGTGTTTAGGAGAAGCTGAATCC
Arid1a-shRNA-mus-1	F: CCGGCAGGCCCTATGGCCCTAATATCTCGAGATATTAGGGCCATAGGGCCTGTTTTTG
	R: AATTCAAAAACAGGCCCTATGGCCCTAATATCTCGAGATATTAGGGCCATAGGGCCTG
Arid1a-shRNA-mus-2	F: CCGGTGCCCAAGATCGAGGTTATATCTCGAGATATAACCTCGATCTTGGGCATTTTTG
	R: AATTCAAAAATGCCCAAGATCGAGGTTATATCTCGAGATATAACCTCGATCTTGGGCA
**Primers for real-time RT-qPCR**	
A20-mus	F: ACTGGAATGACGAATGGGACA R: CAGGGAATTGTACTGAAGTCCAC
Cxcl1-mus	F: ACTGCACCCAAACCGAAGTC R: TGGGGACACCTTTTAGCATCTT
Cxcl2-mus	F: CCAACCACCAGGCTACAGG R: GCGTCACACTCAAGCTCTG
Cxcl3-mus	F: GATTTTGAGACCATCCAGAGC R: CTCTTCAGTATCTTCTTGATG
Cxcl9-mus	F: GGAGTTCGAGGAACCCTAGTG R: GGGATTTGTAGTGGATCGTGC
Cxcl10-mus	F: CCAAGTGCTGCCGTCATTTTC R: GGCTCGCAGGGATGATTTCAA
Cxcl11-mus	F: GTAAACCAGTCAGCCTGAG R: GCTTTCTCCAGGTACTCTTG
Ccl2-mus	F: GGCCTGCTGTTCACAGTTG R: CTGCTGGTGATCCTCTTGTAG
Ccl3-mus	F: CTGCAACCAAGTCTTCTCAG R: GCCGGTTTCTCTTAGTCAGG
Ccl4-mus	F: CTTCTGTGCTCCAGGGTTCTC R: CTGTCTGCCTCTTTTGGTCAG
Ccl5-mus	F: GCTGCTTTGCCTACCTCTCC R: TCGAGTGACAAACACGACTGC
Arid1a-mus	F: CTTCCCCAACCACCAGTACAA R: CTGTGCGAAGGACGAAGAC
Arid1b-mus	F: GTTGTATGGGATGGGTACTCACC R: CTGGTTGTAGTATGGCTGCTG
Ar-mus	F: CTGGGAAGGGTCTACCCAC R: GGTGCTATGTTAGCGGCCTC
Tmprss2-mus	F: CAGTCTGAGCACATCTGTCCT R: CTCGGAGCATACTGAGGCA
Ck8-mus	F: TCCATCAGGGTGACTCAGAAA R: CCAGCTTCAAGGGGCTCAA
Ck18-mus	F: CAGCCAGCGTCTATGCAGG R: CTTTCTCGGTCTGGATTCCAC
Ck5-mus	F: TCTGCCATCACCCCATCTGT R: CCTCCGCCAGAACTGTAGGA
Trp63-mus	F: TACTGCCCCGACCCTTACAT R: GCTGAGGAACTCGCTTGTCTG
Ezh2-mus	F: AGTGACTTGGATTTTCCAGCAC R: AATTCTGTTGTAAGGGCGACC
Sox2-mus	F: GCGGAGTGGAAACTTTTGTCC R: CGGGAAGCGTGTACTTATCCTT
Ascl1-mus	F: GCAACCGGGTCAAGTTGGT R: GTCGTTGGAGTAGTTGGGGG
Brg1-mus	F: CAAAGACAAGCATATCCTAGCCA R: CACGTAGTGTGTGTTAAGGACC
Baf155-mus	F: AGCTAGATTCGGTGCGAGTCT R: CCACCAGTCCAGCTAGTGTTTT
Actl6a-mus	F: GTGTACGGCGGAGATGAAGTT R: GGGAAATCAACCTTAGGGCAGT
Actl6b-mus	F: GCGCTCGTCTTTGACATTGG R: ATTGGTGTCGATGTGGAAAATCT
Brm-mus	F: CTCCTGGACCAATTCTGGGG R: CATCGTTGACAGAGGATGTGAG
Dpf1-mus	F: GAGGCCATCGAGCACTGTC R: CGGGCGGGGTAAGTGTAGA
β-actin-mus	F: GGCTGTATTCCCCTCCATCG R: CCAGTTGGTAACAATGCCATGT
ARID1A-homo	F: CCTGAAGAACTCGAACGGGAA R: TCCGCCATGTTGTTGGTGG
**Primers for sgRNA**	
Arid1a-sgRNA-mus-1	F: CACCGGCAGCTGCGAAGATATCGGG R: AAACCCCGATATCTTCGCAGCTGCC
Arid1a-sgRNA-mus-2	F: CACCGTTCCGGTAAAGATCGTGCAG R: AAACCTGCACGATCTTTACCGGAAC
**Primers for real-time Chip-qPCR**	
A20-mus	F: CAAATCCCTCTACTTACAATCCC R: ACCGAAATCACCTCTGTATGTTC
**Primers for enhancer RNA**	
A20-eRNA-mus	F: TGGCAGGCATTCAGACTACAAAA R: AGAGCTTAAAGCAGGGTAGCAAA
