# Supplementary material for: ARID1A loss induces polymorphonuclear myeloid-derived suppressor cell chemotaxis and promotes prostate cancer progression
Source: Nat Commun. 2022 Nov 26;13:7281. doi: 10.1038/s41467-022-34871-9 (PMC9701216; doi:10.1038/s41467-022-34871-9)
Supplement: Supplementary file 3 — Reporting Summary [file 41467_2022_34871_MOESM3_ESM.pdf]

nature portfolio

Corresponding author(s): Jun Qin

Last updated by author(s): Nov 2, 2022

Reporting Summary

Nature Portfolio wishes to improve the reproducibility of the work that we publish. This form provides structure for consistency and transparency in reporting. For further information on Nature Portfolio policies, see our Editorial Policies and the Editorial Policy Checklist.

Statistics

For all statistical analyses, confirm that the following items are present in the figure legend, table legend, main text, or Methods section.

n/a	Confirmed	
☐	☒	The exact sample size (n) for each experimental group/condition, given as a discrete number and unit of measurement
☐	☒	A statement on whether measurements were taken from distinct samples or whether the same sample was measured repeatedly
☐	☒	The statistical test(s) used AND whether they are one- or two-sided<br>*Only common tests should be described solely by name; describe more complex techniques in the Methods section.*
☒	☐	A description of all covariates tested
☒	☐	A description of any assumptions or corrections, such as tests of normality and adjustment for multiple comparisons
☐	☒	A full description of the statistical parameters including central tendency (e.g. means) or other basic estimates (e.g. regression coefficient) AND variation (e.g. standard deviation) or associated estimates of uncertainty (e.g. confidence intervals)
☐	☒	For null hypothesis testing, the test statistic (e.g. F, t, r) with confidence intervals, effect sizes, degrees of freedom and P value noted<br>*Give P values as exact values whenever suitable.*
☒	☐	For Bayesian analysis, information on the choice of priors and Markov chain Monte Carlo settings
☒	☐	For hierarchical and complex designs, identification of the appropriate level for tests and full reporting of outcomes
☐	☒	Estimates of effect sizes (e.g. Cohen's d, Pearson's r), indicating how they were calculated

Our web collection on statistics for biologists contains articles on many of the points above.

Software and code

Policy information about availability of computer code

Data collection	The Flow cytometric data: Beckman Gallios<br>The CYTOF data: DVS Science<br>The RNASeq/ChIPSeq/ATACSeq data: Illumina Hiseq 2500<br>The qRT-PCR data: QuantStudio 7 Flex Real-Time PCR System
Data analysis	Statistical analysis: GraphPad Prism (ver.8)<br>Flow cytometric analysis: Flowjo (ver.10) or cytobank (2.0)<br>Heatmap analyses of RNASeq data: R package (4.2.0), pheatmap (1.0.12)<br>Profile and heatmap analyses of ChIPSeq and ATACSeq data: deepTools (galaxy)<br>PICARD-MarkDuplicates (Galaxy Version 2.18.2.2)<br>MACS2-callpeak (Galaxy Version 2.1.1.20160309.6)<br>ChIPseeker (Galaxy Version 1.18.0)<br>GSEA (3.0_beta_3)<br>GSVA (1.45.5)<br>DEGseq (1.32.0)

For manuscripts utilizing custom algorithms or software that are central to the research but not yet described in published literature, software must be made available to editors and reviewers. We strongly encourage code deposition in a community repository (e.g. GitHub). See the Nature Portfolio guidelines for submitting code & software for further information.

March 2021

Data

Policy information about availability of data

All manuscripts must include a data availability statement. This statement should provide the following information, where applicable:

- Accession codes, unique identifiers, or web links for publicly available datasets
- A description of any restrictions on data availability
- For clinical datasets or third party data, please ensure that the statement adheres to our policy

All RNA-seq, ChIP-seq and ATAC-seq data generated during this study have been deposited in the Gene Expression Omnibus (GEO) database under accession numbers GSE197688 (https://www.ncbi.nlm.nih.gov/geo/query/acc.cgi?acc=GSE197688). Published datasets used in this study are available through GEO (GSE21032, https://www.ncbi.nlm.nih.gov/geo/query/acc.cgi?acc=GSE21032) or cBioPortal database (SU2C/PCF Dream Team, PNAS 2019, https://www.cbioportal.org/). The remaining data are available within the Article, Supplementary Information or Source Data file. Source data are provided with this paper.

Field-specific reporting

Please select the one below that is the best fit for your research. If you are not sure, read the appropriate sections before making your selection.

☒ Life sciences ☐ Behavioural & social sciences ☐ Ecological, evolutionary & environmental sciences

For a reference copy of the document with all sections, see nature.com/documents/nr-reporting-summary-flat.pdf

Life sciences study design

All studies must disclose on these points even when the disclosure is negative.

Sample size	Experimental sample sizes were determined based on extensive prior experience of identical or similar cell biology or biochemical assays or prior experiments using the GEMMs and xenograft models (see for example DOI: https://doi.org/10.1016/j.ccell.2020.05.022). There are at least 5 mice in pairs in all animal models. The sample size is sufficient for statistical analysis in the reseach project.
Data exclusions	No data were excluded from the analysis.
Replication	Experiments were repeated three times independently with similar results.
Randomization	All samples were allocated in random.
Blinding	The investigations were blinded to group allocation during data collection and analysis.

Reporting for specific materials, systems and methods

We require information from authors about some types of materials, experimental systems and methods used in many studies. Here, indicate whether each material, system or method listed is relevant to your study. If you are not sure if a list item applies to your research, read the appropriate section before selecting a response.

Materials & experimental systems

n/a	Involved in the study	
☐	☒	Antibodies
☐	☒	Eukaryotic cell lines
☒	☐	Palaeontology and archaeology
☐	☒	Animals and other organisms
☐	☒	Human research participants
☒	☐	Clinical data
☒	☐	Dual use research of concern

Methods

n/a	Involved in the study	
☐	☒	ChIP-seq
☐	☒	Flow cytometry
☒	☐	MRI-based neuroimaging

Antibodies

Antibodies used	IHC<br>ARID1A, Sigma-Aldrich, Cat# HPA005456; 1:500<br>ARID1A, Cell Signaling Technology, clone:D2A8U, Cat# 12354S; 1:500<br>CD8, Abcam, clone:EPR10640(2), Cat# ab189926; 1:200<br>CD8,Abcam, clone:EPR20305, Cat# ab209775; 1:200<br>CD15, Abcam, clone:FUT4/815, Cat# ab220182; 1:500<br>Ly6G, BioLegend, clone:1A8, Cat# 127601; 1:500<br>CD4, Abcam, clone:EPR19514, Cat# ab183685; 1:500

P65, Cell Signaling Technology, clone:D14E12, Cat# 8242S; 1:500
Ki67, Cell Signaling Technology, clone:D3B5, Cat# 12202S; 1:500
AR, Santa Cruz Biotechnology, clone:N-20, Cat# SC-816; 1:500
SMAa, Sigma-Aldrich, clone:1A4, Cat# A2547; 1:4000
CK8, Abcam, Cat# ab154301; 1:4000
Biotinylated Goat Anti-Mouse IgG Antibody (H+L) Vector Labs Cat# BA-9200-1.5; 1:500
Biotinylated Goat Anti-Rabbit IgG Antibody (H+L) Vector Labs Cat# BA-1000-1.5; 1:500
ChIP
ARID1A, Cell Signaling Technology, clone:D2A8U, Cat# 12354S;
BRG1, Abcam, clone:EPNCIR111A, Cat# ab110641;
BAF155, Santa Cruz Biotechnology, clone:G-7, Cat# sc-365543X;
ARID1B, Cell Signaling Technology, clone:E9J4T, Cat# 92964S;
H3K27ac, Cell Signaling Technology, clone:D5E4, Cat# 8173S;
H3K4me1, Cell Signaling Technology, clone:D1A9, Cat# 5326;
H3K4me3, Cell Signaling Technology, clone:C42D8, Cat# 9751S;
10 μg antibody for each ChIP reaction
FACS
F4/80-BV510, BioLegend, clone:BM8, Cat# 123135;
CD11b-BV605, BioLegend, clone:M1/70, Cat# 101237;
Ly6G-PE-Cy7, BioLegend, clone:1A8, Cat# 127617;
Ly6C-APC, BioLegend, clone:HK1.4, Cat# 128016;
CD45-APC-Cy7, BioLegend, clone:30-F11, Cat# 103116;
CD4-PE-Cy7, BioLegend, clone:GK1.5, Cat# 100422;
CD8-AF700, BioLegend, clone:53-6.7, Cat# 100730;
FOXP3-APC, eBioscience, clone:236A/E7, Cat# 17-4777;
Ki67-BV421, BioLegend, clone:16A8, Cat# 652411;
IFNγ-FITC, BioLegend, clone:XMG1.2, Cat# 505806;
CD16/CD32, Biolegend, Cat# 156604;
CD24-FITC, BioLegend, clone:M1/69, Cat# 101806;
CD49f-PE, eBioscience, clone:GoH3, Cat# 12-0495-82;
All Facs antiboies were used in a dilution of 1:100.
CYTOF
Ly-6G, conjugated to 141Pr, DVS-Fluidigm, clone:1A8, Cat# 3141008B;
CD4(Ms), conjugated to 145Nd, DVS-Fluidigm, clone:RM4-5, Cat# 3145002B;
CD8a (Ms), conjugated to 146Nd, DVS-Fluidigm, clone:53-6.7, Cat# 3146003B;
CD45 (Ms), conjugated to 147Sm, DVS-Fluidigm, clone:30-F11, Cat# 3147003B;
CD11b (Ms), conjugated to 148Nd, DVS-Fluidigm, clone:M1/70, Cat# 3148003B;
CD3e, conjugated to 152Sm, DVS-Fluidigm, clone:145-2C11, Cat# 3152004B;
F4/80, conjugated to 159Tb, DVS-Fluidigm, clone:BM8, Cat# 3159009B;
Ly-6C, conjugated to 162Dy, DVS-Fluidigm, clone:HK1.4, Cat# 3162014B;
IFNγ, conjugated to 165Ho, DVS-Fluidigm, clone:XMG1.2, Cat# 3165003B;
Western
β-actin, proteintech, clone:2D4H5, Cat# 66009-1-Ig; 1:5000
FLAG, Cell Signaling Technology, clone:D6W5B, Cat# 14793S; 1:1000
HA, Cell Signaling Technology, clone:C29F4, Cat# 3724S; 1:1000
Ubiquitin Cell Signaling Technology Cat# 3933S; 1:2000
P65, Santa Cruz Biotechnology, clone:A-12, Cat# sc-514451; 1:2000
p-P65, Cell Signaling Technology, clone:93H1, Cat# 3033S; 1:2000
IKKα, Cell Signaling Technology, Cat# 2682S; 1:500
IKKβ, Cell Signaling Technology, clone:2C8, Cat# 2370S; 1:1000
NEMO, Abcam, clone:EPR16629, Cat# ab178872; 1:2000
p-IKKα/β, Cell Signaling Technology, clone:16A6, Cat# 2697S; 1:500
p105/p50, Cell Signaling Technology, clone:D7H5M, Cat# 12540S; 1:2000
p100/p52, Cell Signaling Technology, clone:18D10, Cat# 3017S; 1:2000
β-TRCP, Cell Signaling Technology, clone:D13F10, Cat# 4394S; 1:1000
STAT1, Cell Signaling Technology, Cat# 9172S; 1:2000
p-STAT1, Cell Signaling Technology, clone:D4A7, Cat# 7649S; 1:2000
STAT3, Cell Signaling Technology, clone:79D7, Cat# 4904S; 1:2000
p-STAT3, Cell Signaling Technology, clone:D3A7, Cat# 9145S; 1:2000
Anti-Thiophosphate ester antibody, Abcam, clone:51-8, Cat# ab92570; 1:1000
ARD1B, Cell Signaling Technology, clone:E9J4T, Cat#92964; 1:1000
BAF155, Cell Signaling Technology, clone:D7F8S, Cat#11956; 1:2000
BAF53B Abclonal Cat#A7108; 1:2000
BAF45B Abclonal Cat#A7349; 1:2000
Others
InVivoMAb anti-mouse Ly6G (clone 1A8) BioXcell Cat# BE0075-1;
InVivoMAb anti-mouse PD-1 (clone RMP1-14) BioXcell Cat# BE0146;
InVivoMAb anti-mouse CTLA4 (clone 9H10) BioXcell Cat# BE0131;
Cxcl2 R&D Systems Cat# MAB452;

Validation

IHC
ARID1A Sigma-Aldrich Cat# HPA005456; https://www.sigmaaldrich.cn/CN/zh/product/sigma/hpa005456
ARID1A Cell Signaling Technology Cat# 12354S; https://www.cellsignal.com/products/primary-antibodies/arid1a-baf250a-d2a8u-rabbit-mab/12354
CD8 Abcam Cat# ab189926; https://www.abcam.cn/cd8-alpha-antibody-epr106402-n-terminal-ab189926.html

CD8 Abcam Cat# ab209775; https://www.abcam.cn/cd8-alpha-antibody-epr20305-ab209775.html
Ly6G BioLegend Cat# 127601; https://www.biolegend.com/en-us/products/purified-anti-mouse-ly-6g-antibody-4767?GroupID=BLG7232
CD15 Abcam Cat# ab220182; https://www.abcam.cn/cd15-antibody-fut4815-ab220182.html
CD4 Abcam Cat# ab183685; https://www.abcam.cn/cd4-antibody-epr19514-ab183685.html
P65 Cell Signaling Technology Cat# 8242S; https://www.cellsignal.com/products/primary-antibodies/nf-kb-p65-d14e12-xp-rabbit-mab/8242
Ki67 Cell Signaling Technology Cat# 12202S; https://www.cellsignal.com/products/primary-antibodies/ki-67-d3b5-rabbit-mab-mouse-preferred-ihc-formulated/12202?site-search-type=Products&N=4294956287&Ntt=12202s&fromPage=plp&_requestid=3237551
AR Santa Cruz Biotechnology Cat# SC-816; https://www.scbt.com/p/ar-antibody-n-20?requestFrom=search
SMAa Sigma-Aldrich Cat# A2547; https://www.sigmaaldrich.cn/CN/zh/product/sigma/a2547
CK8 Abcam Cat# ab154301; https://www.abcam.com/cytokeratin-8-antibody-ab154301.html
ChIP
ARID1A Cell Signaling Technology Cat# 12354S; https://www.cellsignal.com/products/primary-antibodies/arid1a-baf250a-d2a8u-rabbit-mab/12354
BRG1 Abcam Cat# ab110641; https://www.abcam.com/brg1-antibody-epncir111a-ab110641.html
BAF155 Santa Cruz Biotechnology Cat# sc-365543X; https://www.scbt.com/p/baf155-antibody-g-7?requestFrom=search
ARID1B Cell Signaling Technology Cat# 92964S; https://www.cellsignal.com/products/primary-antibodies/arid1b-baf250b-e9j4t-rabbit-mab/92964?site-search-type=Products&N=4294956287&Ntt=92964s&fromPage=plp&_requestid=3246846
H3K27ac Cell Signaling Technology Cat# 8173S; https://www.cellsignal.com/products/primary-antibodies/acetyl-histone-h3-lys27-d5e4-xp-rabbit-mab/8173?site-search-type=Products&N=4294956287&Ntt=h3k27ac&fromPage=plp
H3K4me Cell Signaling Technology Cat# 5326S; https://www.cellsignal.com/products/primary-antibodies/mono-methyl-histone-h3-lys4-d1a9-xp-rabbit-mab/5326
H3K4me3 Cell Signaling Technology Cat# 9751S; https://www.cellsignal.com/products/primary-antibodies/tri-methyl-histone-h3-lys4-c42d8-rabbit-mab/9751?site-search-type=Products&N=4294956287&Ntt=9751s&fromPage=plp&_requestid=3248235
FACS
F4/80-BV510 BioLegend Cat# 123135; https://www.biolegend.com/en-us/products/brilliant-violet-510-anti-mouse-f4-80-antibody-8934?GroupID=BLG5319
CD11b-BV605 BioLegend Cat# 101237; https://www.biolegend.com/en-us/products/brilliant-violet-605-anti-mouse-human-cd11b-antibody-7637
Ly6G-PE-Cy7 BioLegend Cat# 127617; https://www.biolegend.com/en-us/products/pe-cyanine7-anti-mouse-ly-6g-antibody-6139
Ly6C-APC BioLegend Cat# 128016; https://www.biolegend.com/en-us/products/apc-anti-mouse-ly-6c-antibody-6047
CD45-APC-Cy7 BioLegend Cat# 103116; https://www.biolegend.com/en-us/products/apc-cyanine7-anti-mouse-cd45-antibody-2530
CD3-PE BioLegend Cat# 100245; https://www.biolegend.com/en-us/products/pe-dazzle-594-anti-mouse-cd3-antibody-11946
CD4-PE-Cy7 BioLegend Cat# 100422; https://www.biolegend.com/en-us/products/pe-cyanine7-anti-mouse-cd4-antibody-1919
CD8-AF700 BioLegend Cat# 100730; https://www.biolegend.com/en-us/products/alexa-fluor-700-anti-mouse-cd8a-antibody-3387
FOXP3-APC eBioscience Cat# 17-4777; https://www.thermofisher.cn/cn/zh/antibody/product/FOXP3-Antibody-clone-236A-E7-Monoclonal/17-4777-42
Ki67-BV421 BioLegend Cat# 652411; https://www.biolegend.com/en-us/products/brilliant-violet-421-anti-mouse-ki-67-antibody-8982
IFNγ-FITC BioLegend Cat# 505806; https://www.biolegend.com/en-us/products/fitc-anti-mouse-ifn-gamma-antibody-995
CD16/CD32 Biolegend Cat# 156604; https://www.biolegend.com/en-us/products/trustain-fcx-plus-anti-mouse-cd16-32-antibody-17085
CD24-FITC BioLegend Cat# 101806; https://www.biolegend.com/en-us/products/fitc-anti-mouse-cd24-antibody-341
CD49f-PE eBioscience Cat# 12-0495-82; https://www.thermofisher.cn/cn/zh/antibody/product/CD49f-Integrin-alpha-6-Antibody-clone-eBioGoH3-GoH3-Monoclonal/12-0495-82
CYTOF
Ly-6G conjugated to 141Pr DVS-Fluidigm Cat# 3141008B; https://store.fluidigm.com/Cytometry/ConsumablesandReagentsCytometry/MaxparAntibodies/Anti-Mouse%20Ly-6G%20-1A8-141Pr%E2%80%94100%20Tests?cclcl=en_US
CD4(Ms) conjugated to 145Nd DVS-Fluidigm Cat# 3145002B; https://store.fluidigm.com/Cytometry/ConsumablesandReagentsCytometry/MaxparAntibodies/Anti-Mouse%20CD4%20-RM4-5-145Nd%E2%80%94100%20Tests?cclcl=en_US
CD8a (Ms) conjugated to 146Nd DVS-Fluidigm Cat# 3146003B; https://store.fluidigm.com/Cytometry/ConsumablesandReagentsCytometry/MaxparAntibodies/Anti-Mouse%20CD8a%20-53-6-7-146Nd%E2%80%94100%20Tests?cclcl=en_US
CD45 (Ms) conjugated to 147Sm DVS-Fluidigm Cat# 3147003B; https://store.fluidigm.com/Cytometry/ConsumablesandReagentsCytometry/MaxparAntibodies/Anti-Mouse%20CD45%20-30-F11-147Sm%E2%80%94100%20Tests?cclcl=en_US
CD11b (Ms) conjugated to 148Nd DVS-Fluidigm Cat# 3148003B; https://store.fluidigm.com/Cytometry/ConsumablesandReagentsCytometry/MaxparAntibodies/Anti-Mouse%20CD11b%20-M1-70-148Nd%E2%80%94100%20Tests?cclcl=en_US
CD3e conjugated to 152Sm DVS-Fluidigm Cat# 3152004B; https://store.fluidigm.com/Cytometry/ConsumablesandReagentsCytometry/MaxparAntibodies/Anti-Mouse%20CD3e%20-145-2C11-152Sm%E2%80%94100%20Tests?cclcl=en_US
F4/80 conjugated to 159Tb DVS-Fluidigm Cat# 3159009B; https://store.fluidigm.com/Cytometry/ConsumablesandReagentsCytometry/MaxparAntibodies/Anti-Mouse%20F4-80%20-BM8-159Tb%E2%80%94100%20Tests?cclcl=en_US
Ly-6C conjugated to 162Dy DVS-Fluidigm Cat# 3162014B; https://store.fluidigm.com/Cytometry/ConsumablesandReagentsCytometry/MaxparAntibodies/Anti-Mouse%20Ly-6C%20-HK1-4-162Dy%E2%80%94100%20Tests?cclcl=en_US
IFNγ conjugated to 165Ho DVS-Fluidigm Cat# 3165003B; https://store.fluidigm.com/Cytometry/ConsumablesandReagentsCytometry/MaxparAntibodies/Anti-Mouse%20IFNg%20-XMG1-2-165Ho%E2%80%94100%20Tests?cclcl=en_US
Western
β-actin proteintech Cat# 66009-1-Ig; https://www.ptglab.com/products/Pan-Actin-Antibody-66009-1-Ig.htm

FLAG Cell Signaling Technology Cat# 14793S; https://www.cellsignal.com/products/primary-antibodies/dykddddk-tag-d6w5b-rabbit-mab-binds-to-same-epitope-as-sigma-s-anti-flag-m2-antibody/14793
HA Cell Signaling Technology Cat# 3724S; https://www.cellsignal.com/products/primary-antibodies/ha-tag-c29f4-rabbit-mab/3724
Ubiquitin Cell Signaling Technology Cat# 3933S; https://www.cellsignal.com/product/productDetail.jsp?productId=3933
P65 Santa Cruz Biotechnology Cat# sc-514451; https://www.scbt.com/p/nfkappab-p65-antibody-a-12?requestFrom=search
p-P65 Cell Signaling Technology Cat# 3033S; https://www.cellsignal.com/products/primary-antibodies/phospho-nf-kb-p65-ser536-93h1-rabbit-mab/3033?site-search-type=Products&N=4294956287&Ntt=3033s&fromPage=plp&_requestid=3252314
IKKα Cell Signaling Technology Cat# 2682S; https://www.cellsignal.com/products/primary-antibodies/ikka-antibody/2682?site-search-type=Products&N=4294956287&Ntt=2682p&fromPage=plp&_requestid=3252371
IKKβ Cell Signaling Technology Cat# 2370S; https://www.cellsignal.com/products/primary-antibodies/ikkb-2c8-rabbit-mab/2370?site-search-type=Products&N=4294956287&Ntt=2370s&fromPage=plp&_requestid=3295078
NEMO Abcam Cat# ab178872; https://www.abcam.com/ikk-gammanemo-antibody-epr16629-ab178872.html
p-IKKα/β Cell Signaling Technology Cat# 2697S; https://www.cellsignal.com/products/primary-antibodies/phospho-ikka-b-ser176-180-16a6-rabbit-mab/2697?site-search-type=Products&N=4294956287&Ntt=2697s&fromPage=plp&_requestid=3252522
P50 Cell Signaling Technology Cat# 12540S; https://www.cellsignal.com/products/primary-antibodies/nf-kb1-p105-p50-d7h5m-rabbit-mab/12540?site-search-type=Products&N=4294956287&Ntt=12540s&fromPage=plp&_requestid=3252739
P52 Cell Signaling Technology Cat# 3017S; https://www.cellsignal.com/products/primary-antibodies/nf-kb2-p100-p52-18d10-rabbit-mab/3017?site-search-type=Products&N=4294956287&Ntt=3017s&fromPage=plp&_requestid=3252788
β-TRCP Cell Signaling Technology Cat# 4394S; https://www.cellsignal.com/products/primary-antibodies/b-trcp-d13f10-rabbit-mab/4394?site-search-type=Products&N=4294956287&Ntt=4394s&fromPage=plp&_requestid=3252852
STAT1 Cell Signaling Technology Cat# 9172S; https://www.cellsignal.com/products/primary-antibodies/stat1-antibody/9172?site-search-type=Products&N=4294956287&Ntt=9172s&fromPage=plp&_requestid=3252911
p-STAT1 Cell Signaling Technology Cat# 7649S; https://www.cellsignal.com/products/primary-antibodies/phospho-stat1-tyr701-d4a7-rabbit-mab/7649?site-search-type=Products&N=4294956287&Ntt=7649p&fromPage=plp&_requestid=3252980
STAT3 Cell Signaling Technology Cat# 4904S; https://www.cellsignal.com/products/primary-antibodies/stat3-79d7-rabbit-mab/4904?site-search-type=Products&N=4294956287&Ntt=4904s&fromPage=plp&_requestid=3253045
p-STAT3 Cell Signaling Technology Cat# 9145S; https://www.cellsignal.com/products/primary-antibodies/phospho-stat3-tyr705-d3a7-xp-rabbit-mab/9145?site-search-type=Products&N=4294956287&Ntt=9145p&fromPage=plp&_requestid=3253116
Anti-Thiophosphate ester antibody Abcam Cat# ab92570; https://www.abcam.com/thiophosphate-ester-antibody-51-8-ab92570.html
ARID1B Cell Signaling Technology Cat# 92964S; https://www.cellsignal.com/products/primary-antibodies/arid1b-baf250b-e9j4t-rabbit-mab/92964?site-search-type=Products&N=4294956287&Ntt=92964s&fromPage=plp&_requestid=3246846
BAF155 Cell Signaling Technology Cat#11956; https://www.cellsignal.com/products/primary-antibodies/smarcc1-baf155-d7f8s-rabbit-mab/11956
BAF53B Abclonal Cat#A7108 https://ap.abclonal.com/catalog-antibodies/ACTL6BPolyclonalAntibody/A7108
BAF45B Abclonal Cat#7349 https://abclonal.com/Datasheet/Antibodies/A7349.pdf
Others
InVivoMAb anti-mouse Ly6G/Ly6C (clone RB6-8C5) BioXcell Cat# BE0075; https://bxcell.com/product/m-ly-6g-2/
InVivoMAb anti-mouse PD-1 (clone RMP1-14) BioXcell Cat# BE0146; https://bxcell.com/product/invivomab-anti-m-pd-1/
InVivoMAb anti-mouse CTLA4 (clone 9H10) BioXcell Cat# BE0131; https://bxcell.com/product/m-cd152-m-ctla-4/
InVivoMAb rat IgG2a isotype control (clone 2A3) BioXcell Cat# BE0089; https://bxcell.com/product/rat-igg2a-isotype-control/
Cxcl2 R&D Systems Cat# MAB452; https://www.rndsystems.com/cn/products/mouse-cxcl2-grobeta-mip-2-cinc-3-antibody-40605_mab452

Eukaryotic cell lines

Policy information about cell lines

Cell line source(s)	293T ATCC Cat #CRL-11268;<br>C4-2 ATCC Cat# CRL-3314;<br>PC-3 ATCC Cat# CRL-1435;<br>22RV-1 ATCC Cat# CRL-2505;<br>DU145 ATCC Cat# HTB-81;<br>A549 Cell Bank, Shanghai Institute of Biochemistry and Cell Biology (SIBCB), Chinese Academy of Sciences Cat# SCSP-503;<br>HCT116 ATCC Cat# CCL-247;<br>Jurkat Cell Bank, Shanghai Institute of Biochemistry and Cell Biology (SIBCB), Chinese Academy of Sciences Cat# SCSP-513;<br>RAW 264.7 ATCC Cat# TIB-71;<br>Myc-CaP ATCC Cat# CRL-3255;<br>OCI-LY10 Nanjing Cobioer Biosciences Co., LTD Cat# CBP60558;<br>Pten; Trp53; Smad4 triple KO (TKO) cells isolated from ProbasinCre/+; Trp53flox/flox; Smad4flox/flox mouse with PCa
Authentication	All cell lines were authenticated by observing the morphology.
Mycoplasma contamination	There is no mycoplasma contamination in all cell lines.
Commonly misidentified lines<br>(See ICLAC register)	No commonly misidentified cell lines were used.

Animals and other organisms

Policy information about studies involving animals; ARRIVE guidelines recommended for reporting animal research

Laboratory animals	Arid1a-floxed mice were provided by Dr. Hongbin Ji (Shanghai Institute of Biochemistry and Cell Biology, Chinese Academy of Sciences). Pten-floxed mice were generated by Hong Wu. The PBCre/+ transgenic mice were obtained from Fen Wang. For GEMM

	studies, all mice are maintained in C57BL/6 background. Male mice were sacrificed for analysis at 3- or 4-month-old. For subcutaneous injection, 4- to 6-week-old male mice (C57BL/6, FVB or Rag1−/− mice; Shanghai SLAC Laboratory Animal Co., Ltd) were injected with 1×106 cells. 10-week-old PtenPC−/− and PtenPC−/−; Arid1aPC−/− mice received castration. Two weeks later, Enzalutamide was given 3 times a week for 2 months. More details about the number and age of animals used in each experiment, see the corresponding figure legends.<br>Mice were housed under specific-pathogen-free conditions with standard food and water ad libitum in a 12h light / 12h dark cycle. Humidity and ambient temperature were maintained between 45-65% and 20-24°C, respectively.
Wild animals	The study did not involve wild animals.
Field-collected samples	The study did not involve sample collected from the filed.
Ethics oversight	Mouse experimental protocols were approved by the Institutional biomedical research ethics committee of Shanghai Institute of Nutrition and Health Sciences, Chinese Academy of Sciences.

Note that full information on the approval of the study protocol must also be provided in the manuscript.

Human research participants

Policy information about studies involving human research participants

Population characteristics	Clinical summary of tissue microarray from PCa patients was listed in Supplementary Table 1. Clinical summary of tumor tissue specimens from advanced PCa patients was listed in Supplementary Table 2.
Recruitment	For tissue microarry, PCa patients who underwent radical prostatectomy surgery between 2012/08 - 2020/03 were included. Both tumor (Gleason score ≥ 7) and Benign Prostatic Hyperplasia were collected. For tumor tissue specimens, PCa patients with high-risk (Gleason score at initial diagnosis ≥ 7), localized disease who underwent radical prostatectomy surgery were included.
Ethics oversight	The use of pathological specimens, as well as the review of all pertinent patient records, was approved by the ethical standards of the institutional research committee and with the 1964 Declaration of Helsinki and its later amendments or comparable ethical standards. All patient samples were collected by the Department of Pathology with approval from the Research Ethics Committee of Daping Hospital, Army Medical University, and informed consent was obtained from the patients.

Note that full information on the approval of the study protocol must also be provided in the manuscript.

ChIP-seq

Data deposition

☒ Confirm that both raw and final processed data have been deposited in a public database such as GEO.

☒ Confirm that you have deposited or provided access to graph files (e.g. BED files) for the called peaks.

Data access links<br>*May remain private before publication.*	GSE197688<br>https://www.ncbi.nlm.nih.gov/geo/query/acc.cgi?acc=GSE197688
Files in database submission	WT-Brg1.bigwig<br>WT-H3K27ac.bigwig<br>KO-Brg1.bigwig<br>KO-H3K27ac.bigwig<br>input.bigwig<br>WT-H3K4me.bigwig<br>WT-H3K4me3.bigwig<br>input-2.bigwig<br>WT_Brg1.read1_Clean.fastq.gz<br>WT_Brg1.read2_Clean.fastq.gz<br>WT_K27.read1_Clean.fastq.gz<br>WT_K27.read2_Clean.fastq.gz<br>KO_Brg1.read1_Clean.fastq.gz<br>KO_Brg1.read2_Clean.fastq.gz<br>KO_K27.read1_Clean.fastq.gz<br>KO_K27.read2_Clean.fastq.gz<br>input.read1_Clean.fastq.gz<br>input.read2_Clean.fastq.gz<br>H3K4me.read1_Clean.fastq.gz<br>H3K4me.read2_Clean.fastq.gz<br>H3K4me3.read1_Clean.fastq.gz<br>H3K4me3.read2_Clean.fastq.gz<br>Input-2.read1_Clean.fastq.gz<br>Input-2.read2_Clean.fastq.gz

Genome browser session (e.g. UCSC)	No longer applicable

Methodology

Replicates	ChIP-seq for each group of the cells was performed once.
Sequencing depth	The amount of data for each sample is 6G,Reads were aligned to the mouse genome (mm10), and after removal of duplicate and non-uniquely mapped reads, ~8 million alignments were obtained.They are paired-end.
Antibodies	BRG1 Abcam Cat# ab110641;<br>H3K27ac Cell Signaling Technology Cat# 8173S;<br>H3K4me Cell Signaling Technology Cat# 5326S;<br>H3K4me3 Cell Signaling Technology Cat# 9751S;
Peak calling parameters	Reads mapped to the same genomic positions were filtered and the nonredundant reads were used for peak calling. MACS2-callpeak was used for performing peak calling with the threshold, p value ≤ 0.005.
Data quality	The BRG1(ab110641,Abcam), H3K27ac (8173S, Cell signaling technology), H3K4me(5326S,Cell signaling technology) and H3K4me3 (9751S, Cell signaling technology) antibodies for ChIP-seq were validated by RiboBio. The correct size of chromatin and libraries were checked in a gel. Only raw reads passing the QC were used for alignment.
Software	mapping by BWA-MEM (Galaxy Version 0.7.17.2)<br>filtered by PICARD-MarkDuplicates (Galaxy Version 2.18.2.2)<br>peak calling by MACS2-callpeak (Galaxy Version 2.1.1.20160309.6)<br>peak profile plots and read-density heat maps were generated using deepTools.<br>cistrome overlap analyses were carried out using the ChIPseeker (Galaxy Version 1.18.0).

Flow Cytometry

Plots

Confirm that:

☒ The axis labels state the marker and fluorochrome used (e.g. CD4-FITC).

☒ The axis scales are clearly visible. Include numbers along axes only for bottom left plot of group (a 'group' is an analysis of identical markers).

☒ All plots are contour plots with outliers or pseudocolor plots.

☒ A numerical value for number of cells or percentage (with statistics) is provided.

Methodology

Sample preparation	Tissues were dissected, minced into small pieces and further digested by 1 mg/ml Collagenase Type II, 1 mg/ml Collagenase Type IV and 0.1 mg/ml DNase I recombinant at 37 °C for 30–60 min. One million cells were incubated with 1 µl CD16/CD32 antibody to block the Fc receptor at 4 °C for 10 min. Cell suspension were incubated with cell surface antibodies at 4 °C for 30 min. For intracellular cytokine staining, cells were stimulated for 4 h at 37°C with cell activation cocktail. After permeabilized with fixation/permeabilization buffer solution according to the manufacturer's protocol, cell suspension was incubated with IFNγ antibody at 4 °C for 30 min. For intracellular staining, cells were permeabilized using Foxp3 Fixation/ Permeabilization Buffer for 1 hour at room temperature, protected from light, and then incubated with Ki67, FOXP3. Then, cells were analyzed on a Gallios analyzer.
Instrument	Data collection was performed on a Beckman Gallios flow cytometer.
Software	Analyses were performed by Flowjo or cytobank.
Cell population abundance	At least 200,000 total cells were captured in all experiments.
Gating strategy	For all expreiments, cells were gated by FSC/SSC to exclude debris, followed by gating FSC-A and FSC-H to eliminate nonsinglets. Then target cell population for further analysis were gated by cell surface markers.

☒ Tick this box to confirm that a figure exemplifying the gating strategy is provided in the Supplementary Information.
